# Identification of genetic variants associated with a wide spectrum of phenotypes clinically diagnosed as Sanfilippo and Morquio syndromes using whole genome sequencing

**DOI:** 10.3389/fgene.2023.1254909

**Published:** 2023-09-11

**Authors:** Rutaba Gul, Sabika Firasat, Mikkel Schubert, Asmat Ullah, Elionora Peña, Anne C. B. Thuesen, Annete P. Gjesing, Mulazim Hussain, Muhammad Tufail, Muhammad Saqib, Kiran Afshan, Torben Hansen

**Affiliations:** ^1^ Department of Zoology, Faculty of Biological Sciences, Quaid-i-Azam University, Islamabad, Pakistan; ^2^ Novo Nordisk Foundation Center for Basic Metabolic Research, Faculty of Health and Medical Sciences, University of Copenhagen, Copenhagen, Denmark; ^3^ The Children Hospital, Pakistan Institute of Medical Sciences (PIMS), Islamabad, Pakistan; ^4^ Department of Zoology, University of Lakki Marwat, Lakki Marwat, Khyber Pakhtunkhwa, Pakistan

**Keywords:** Sanfilippo syndrome, Morquio syndrome, Pakistani families, whole genome sequencing, *VWA3B*

## Abstract

Mucopolysaccharidoses (MPSs) are inherited lysosomal storage disorders (LSDs). MPSs are caused by excessive accumulation of mucopolysaccharides due to missing or deficiency of enzymes required for the degradation of specific macromolecules. MPS I-IV, MPS VI, MPS VII, and MPS IX are sub-types of mucopolysaccharidoses. Among these, MPS III (also known as Sanfilippo) and MPS IV (Morquio) syndromes are lethal and prevalent sub-types. This study aimed to identify causal genetic variants in cases of MPS III and MPS IV and characterize genotype-phenotype relations in Pakistan. We performed clinical, biochemical and genetic analysis using Whole Genome Sequencing (WGS) in 14 Pakistani families affected with MPS III or MPS IV. Patients were classified into MPS III by history of aggressive behaviors, dementia, clear cornea and into MPS IV by short trunk, short stature, reversed ratio of upper segment to lower segment with a short upper segment. Data analysis and variant selections were made based on segregation analysis, examination of known MPS III and MPS IV genes, gene function, gene expression, the pathogenicity of variants based on ACMG guidelines and *in silico* analysis. In total, 58 individuals from 14 families were included in the present study. Six families were clinically diagnosed with MPS III and eight families with MPS IV. WGS revealed variants in MPS-associated genes including *NAGLU, SGSH, GALNS, GNPTG* as well as the genes *VWA3B*, *BTD*, and *GNPTG* which have not previously associated with MPS. One family had causal variants in both *GALNS* and *BTD*. Accurate and early diagnosis of MPS in children represents a helpful step for designing therapeutic strategies to protect different organs from permanent damage. In addition, pre-natal screening and identification of genetic etiology will facilitate genetic counselling of the affected families. Identification of novel causal MPS genes might help identifying new targeted therapies to treat LSDs.

## Introduction

### Background

Mucopolysaccharidoses (MPS) is a group of disorders belonging to lysosomal storage disorders. MPS are sub categorized into seven types (MPS I, II, III, IV, VI, VII, and IX) mostly inherited as autosomal recessive except for MPS II. Each type is caused by the deficiency of mucopolysaccharide-degrading enzymes. Due to the deficiency of these enzymes, the mucopolysaccharides (glycosaminoglycans) accumulate in different organs of the body including the arteries, skeleton, eyes, joints, ears, skin, teeth, respiratory system, liver, spleen, central nervous system, blood, and bone marrow. This excessive accumulation of mucopolysaccharides result in phenotypes of MPS overlapping with one another, making it difficult to diagnose without use of specific diagnostic approaches like the measurement of enzyme activity in leukocytes or molecular diagnosis (Besley and Wraith, 1997; Chih-Kuang et al., 2002).

Mucopolysaccharidosis type III (MPS III) or Sanfilippo syndrome is a subtype of MPS discovered 50 years ago with an autosomal recessive mode of inheritance. The disease affects 0.3–4.1 cases per 100,000 births (Sanfilippo, 1963; Valstar et al., 2008; Fedele, 2015; Khan et al., 2017). It has been further classified into four categories (MPS type IIIA—D) caused by variants in *SGSH*, *NAGLU, HGSNAT*, and *GNS* respectively. Enzymes encoded by these genes are involved in the degradation of a linear polysaccharide known as heparan sulfate (HS). HS has a crucial role in the development of central nervous system (CNS) which explains why patients suffering from Sanfilippo syndrome develop dementia from early childhood (De Pasquale and Pavone, 2019) and have neurological manifestations such as delayed behavioral and developmental milestones starting from early years of life (Shapiro et al., 2017; Ozkinay et al., 2021).

Mucopolysaccharidosis type IV, also known as Morquio syndrome, was first discovered by a pediatrician Luis Morquio in 1929. It has an autosomal recessive mode of inheritance (Morquio, 1929). It is a rare disorder with prevalence of 1 in 40,000 live births (Nelson et al., 2003). There are two types of Morquio syndrome (type A and type B) caused by pathogenic variants in *GALNS* and *GLB1,* respectively. The gene *GALNS* encodes the GALNS enzyme which has a role in the degradation of glycosaminoglycan including chondroitin-6-sulfate (C6S) and keratin sulfate (KS), while GLB1 encoded by *GLB1* is involved in the degradation of keratin sulfate only (Celik et al., 2021). The GLB1 enzyme is also involved in the hydrolyzation of terminal beta galactosyl residues of GM1 gangliosides and glycoproteins (Okada and John, 1968).

Diagnosis of MPS III involves either urinary HS excretion measurement or enzyme measurement from dry blood spot through tandem mass spectrometry or fluorimetry (Seker Yilmaz et al., 2021). The diagnosis of MPS IV involves radiological findings and urinary excretion or liquid chromatography–mass spectrometry (LC-MS) or MS-based method for KS measurements in plasma or urine (Piraud et al., 1993; Martell et al., 2011; Peracha et al., 2018; Chien et al., 2020; Lin et al., 2020). Due to enzymatic pseudo-deficiency, molecular genetic screening of the patients affected with MPS III or MPS IV is the most accurate method to diagnose any subtype of these disorders.

To the best of our knowledge, no prior molecular genetics research has been conducted in Pakistan regarding Sanfilippo cases. However, various international research groups have published studies highlighting different genetic variations found in cases of Pakistani descent across the globe. Notably, the literature includes instances of novel or previously reported variants of Sanfilippo syndrome originating from Pakistani patients. These include a unique deletion that triggers Sanfilippo syndrome type D, specifically the c.1169delA mutation within exon 10, resulting in a premature termination codon (Beesley et al., 2003). Additionally, a new nonsense variant associated with Sanfilippo syndrome type C, identified as p.Ser296Ter in exon 10, as well as a splice site variant designated c.744–2A>G in intron 7, have been documented (Feldhammer et al., 2009a; Feldhammer et al., 2009b). Several studies have been conducted in Pakistan related to cases of Morquio syndrome, revealing a total of seven new variants—namely, p. Phe216Ser, p. Met38Arg, p. Ala291Ser, p. Glu121Argfs*37, p. Tyr294Terfs—alongside two previously reported variants, p. Pro420Arg and p. Arg386Cys (Tomatsu et al., 2004; Morrone et al., 2014; Ullah et al., 2017; Zubaida et al., 2018).

Treatment options for MPS III and IV include enzyme replacement therapy (ERT) (MPS IV A), molecular chaperon therapy (MCT), substrate reduction therapy (SRT), hematopoietic stem cell transplant (HSCT), *in vivo* adeno-associated viral gene therapy, antioxidant therapy, inhibiting protein aggregation, stop-codon readthrough therapy and anti-inflammatory therapy (Hendriksz et al., 2013; Akyol et al., 2019; Beneto et al., 2020; Seker Yilmaz et al., 2021). In a recent study, recombinant human GALNS enzyme (rhGALNS) was infused with hydrogel like polyethylene glycol to deliver the exogenous enzyme successfully into the fibroblasts for sustainable and longer release (Jain et al., 2020). Molecular genetic testing, prenatal screening, carrier testing, and genetic counselling can help in the prevention of the disease and/or saving organs from permanent degeneration by allowing early diagnosis and appropriate treatment options (where available). This study was designed to evaluate the clinical profiles of Sanfilippo and Morquio syndromes patients from Pakistan, to identify causal gene variants using WGS and to correlate the genotypes with phenotypes.

## Materials and methodology

### Ethical approval and patient recruitment

The study was approved by the bioethical committee of Quaid-i-Azam University (BEC-FBS-QAU2019-198), Islamabad, Pakistan.

**FIGURE 1:**
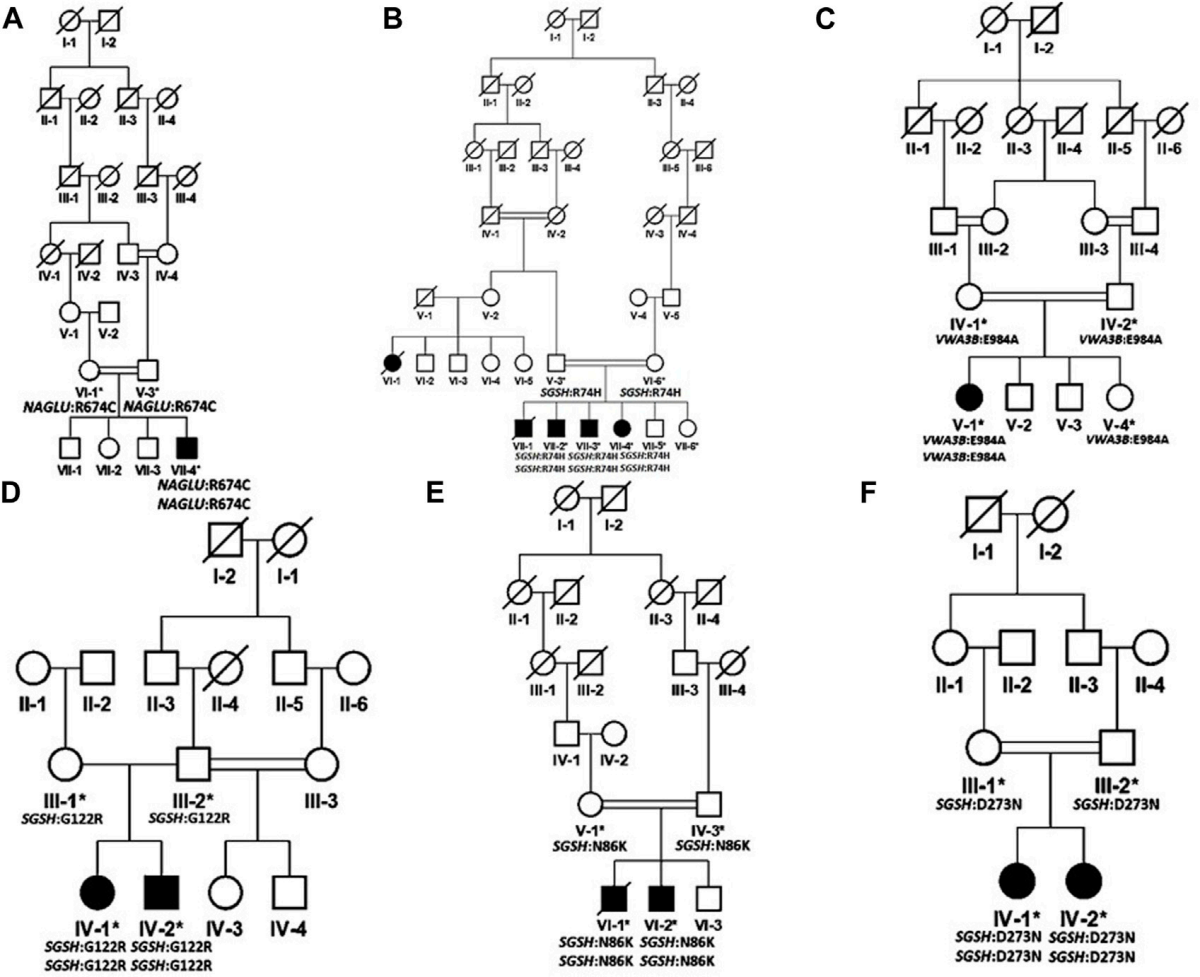
Pedigrees of families inheriting MPS III phenotypes **(A–F)** consistent with autosomal recessive mode of inheritance. Square and circles denote males and females respectively; filled symbols indicate affected individuals and consanguinity is represented by double marriage lines.

**FIGURE 2:**
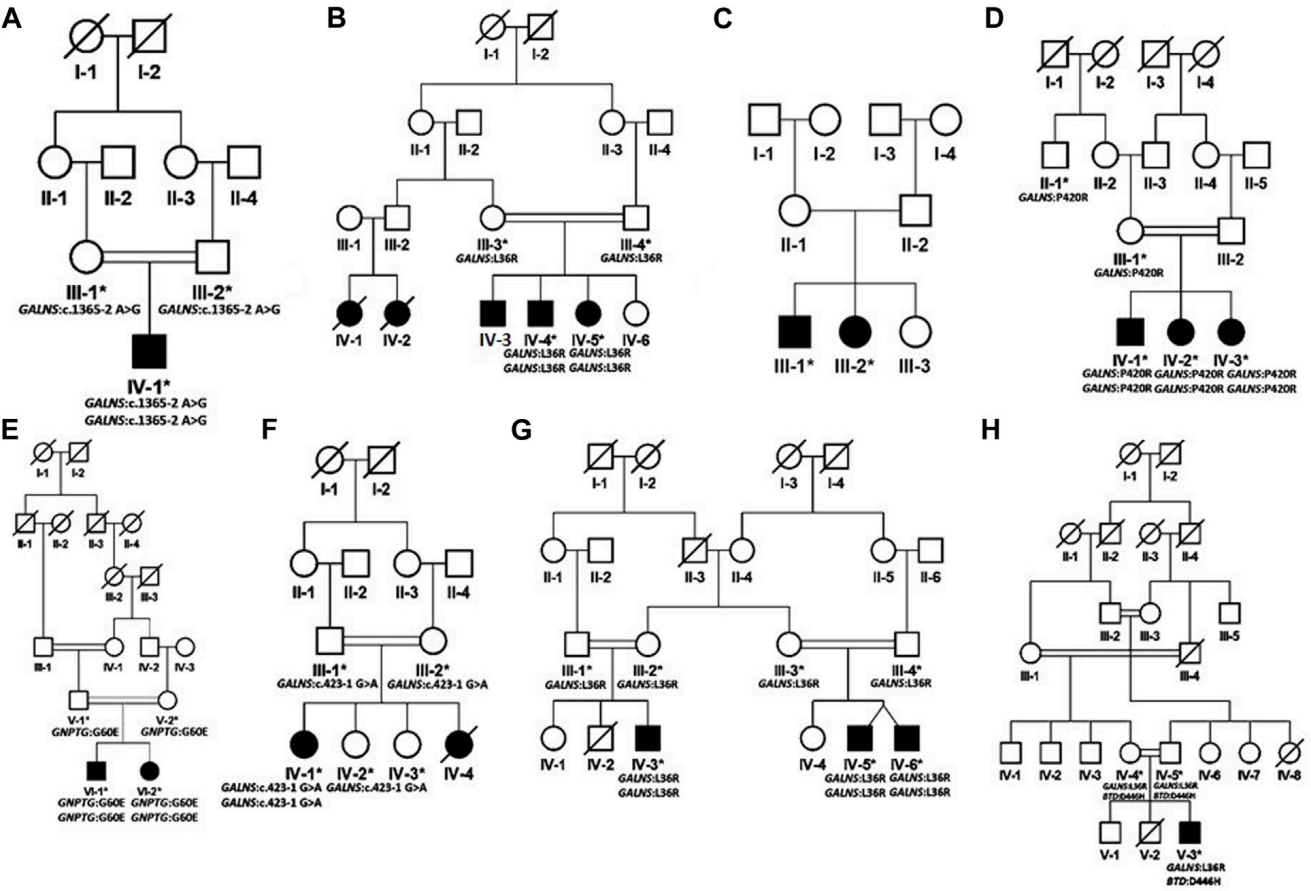
Pedigrees of families inheriting MPS IV phenotypes **(A–H)** consistent with autosomal recessive mode of inheritance. Square and circles denote males and females respectively; filled symbols indicate affected individuals and consanguinity is represented by double marriage lines.

**FIGURE 3:**
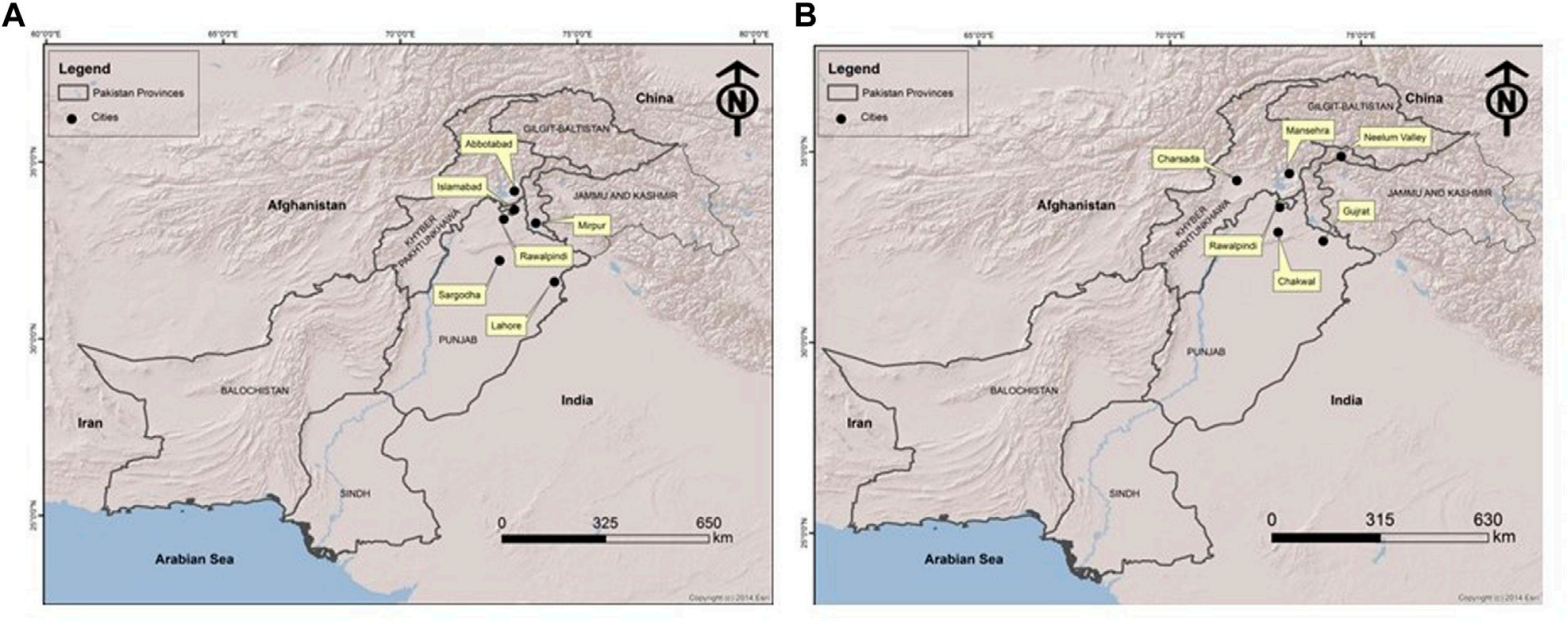
Map of Pakistan showing the locations (circles) of cities/towns from where families included in this study were collected. **(A)** All MPS III collected families. **(B)** All MPS IV collected families.

Patients were recruited and diagnosed based on their presenting clinical profiles (Table 1; Table 3) at outpatient department (OPD), Children Hospital, Pakistan Institute of Medical Sciences (PIMS), Islamabad, Pakistan. Before collecting blood samples from patients and accompanying parents/siblings, informed written consent forms were signed by the participants in accordance with the Declaration of Helsinki (World Medical Association, 2013). The detailed pedigrees were drawn using the information provided by well-informed elders of the family (Figure 1; Figure 2). Localities of families from different regions of Pakistan is mentioned in Figure 3.

**TABLE 1:** Clinical details of the patients suspected as Sanfilippo syndrome affected.

Pedigree no.	Individual no.	No. of affected individuals	Age/Sex	Cast/Region/Consanguinity	Phenotypes/Clinical profile
**A**	VII-4	1	5 y/M	Sardar/AJK/Yes	Delay in achieving milestones, abdominal distention, aggressive behavior (sometimes), speech delay, short stature, increased body hair, coarse facial features, broad based gait, joint contracture, short trunk, hepatosplenomegaly, self-biting, intellectual disability ALT = 49 U/L (up to 40), AST = 46 U/L (up to 40), Bilirubin total = 1.9 mg/dL (<1.2), Hb = 11.1 g/dL (12–1.4), RBC count = 4.08 mil/cmm (4.75–4.85), Hematocrit = 34% (36–42), Monocytes = 06% (4–5), Serum Sodium = 155 mmol/L (132–145), Serum Potassium = 5.5 mmol/L (3.1–5.1), Serum Ferritin = 27.84 ng/mL (30–400), beaking at L1 and L2 vertebrae, Glucose (R) = 76 mg/dL (80–130)
**B**	VII-2	5	14.5 y/M	Khawaja/Punjab/Yes	Delay in achieving milestones, chronic diarrhea, abdominal distention, aggressive behavior, few words speech only, hernia (surgery), short stature, constipation, coarse facial features, hepatosplenomegaly, low IQ
	VII-3		9 y/M		Delay in achieving milestones, chronic diarrhea, abdominal distention, aggressive behavior, few words speech only, hernia (surgery), short stature, constipation, coarse facial features, hepatosplenomegaly, low IQ
	VII-4		10.5 y/F		Delay in achieving milestone, chronic diarrhea, abdominal distention, aggressive behaviors, few words speech only, hernia (surgery), short stature, constipation, coarse facial features, hepatosplenomegaly, low IQ Hb = 9.9 g/dL (13.5–19.5), RBC = 3.92×10^6^/ul (4.0–5.9), MCH = 25.2 pg (28–32), MCHC = 27.7 g/dL (29–37), Neutrophils = 76.1% (50–75), Eosinophils = 0.1% (1–6)
**C**	V-1	2	5 y/F	Aryn/Punjab/Yes	Delay in achieving milestones, chronic diarrhea, aggressive behavior, speech delay, short stature, coarse facial features, joint contractures, hepatosplenomegaly, intellectual disability
	V-4		1.5 y/F		Delay in achieving milestones, chronic diarrhea, short stature, joint contractures, hepatosplenomegaly, intellectual disability
**D**	IV-2	2	1 y/M	Qaisrani Baloch/ICT/No	Delay in achieving milestones, abdominal distention, aggressive behavior, speech delay, short stature, respiratory and ear infection (Otitis media), coarse facial features, joint contractures, hepatosplenomegaly, intellectual disability Mild cerebral atrophy, mild mucosal thickness of right ethmoid and right maxillary sinus, Urea = 10 mg/dL (15–45), Creatinine = 0.5 mg/dL (0.7–1.4), Uric acid = 2.8 md/dL (3.6–7.7), Serum Ferritin = 4.75 μg/L (30–400), Hb = 10.1 g/dL (14–18), HCT = 36.5% (41–51), MCV = 69.4 fL (80.97), MCH = 19.2 pg (26–32), MCHC = 27.7 g/dL (32–36), Microcytes = Positive, Hypochromia = Positive, Poikilocytes = Positive
	IV-1		2 y/F		Delay in achieving milestones, abdominal distention, aggressive behavior, speech delay, short stature, respiratory and ear infection, coarse facial features, joint contractures, hepatosplenomegaly, intellectual disability 3rd and 4th ventricle dilation, Mild periventricular ischemia, Wide cisterna magna, Mild cerebral atrophy, Urea = 12 mg/dL (15–45), Creatinine = 0.5 g/dL (0.6–1.1), ALT = 39 u/L (5–31), Hb = 10.1 g/dL (12–16), HCT = 35.7% (37–47), MCV = 67.2 fL (80–97), MCH = 19 pg (26–32), MCHC = 28.3 g/dL (32–36), Microcytes = Positive, Hypochromia = Positive, Poikilocytes = Positive
**E**	VI-1	2	14 y/M	Pathan/KPK/Yes	Delay in achieving milestone, aggressive behavior, speech delay, gibbus formation, short stature, respiratory/ear infection, coarse facial features, excessive hair growth, broad based gait/waddling, joint contractures, short stature, scoliosis/kyphosis, intellectual disability Diffuse cerebral cortical atrophy, AV and VA concordance, Dolichocephaly, Mild atrophy, Hb = 11.4 g/dL (14–18), Neutrophils = 44% (50–75), Lymphocytes = 49% (20–40), Monocytes = 01% (2–6)
	VI-2		8 y/M		Delay in achieving milestone, aggressive behavior, speech delay, gibbus formation, short stature, respiratory/ear infection, coarse facial features, excessive hair growth, broad based gait/waddling, joint contractures, short stature, scoliosis/kyphosis, intellectual disability Small acquired porencephalic cyst left peri-sylvian mid parietal region, Hb = 11 g/dL (14–18), WBCs = 3900/cmm (4000–11000), Lymphocytes = 47% (20–45), Monocytes = 01% (2–6)
**F**	IV-1	2	10 y/F	Kaka khel/Punjab/Yes	Delay in achieving milestones, abdominal distention, aggressive behavior, speech delay, respiratory/ear infection (decreased hearing), coarse facial features, joint contractures, hepatosplenomegaly, intellectual disability Spina bifida S1, Irregular acetabular margins, Pallor = Positive, Thick calvarium
	IV-2		7 y/F		Delay in achieving milestones, abdominal distention, aggressive behavior, speech delay, respiratory/ear infection, coarse facial features, joint contractures, hepatosplenomegaly, intellectual disability Hb = 11.7 g/dL (12–15), Neutrophils = 16.7% (60–70), Eosinophils = 25.2% (0.0–4.0), Irregular acetabular with small lytic areas with small femoral epiphysis, Spina bifida S1, Thick calvarium, J-shaped sella, Hip joint juvenile arthritis

AJK, azad jammu and kashmir; ALT, alanine transaminase; AST, aspartate aminotransferase; AV/VA, Atrioventricular/Ventriculoarterial; F*, father; F, female; Hb, Hemoglobin; HCT, hematocrit; ICT, islamabad capital territory; IQ, intelligent quotient; KPK, khyber pakhtunkhwa; M, male; MCV, mean corpuscular volume; MCHC, mean corpuscular hemoglobin concentration; MCH, mean corpuscular hemoglobin; M*, mother; P, patient; RBC, red blood cells; S, sibling; U, uncle; WBCs, White blood cells; y, years.

In presenting clinical profiles, height of each MPS IV patient was measured and compared to the healthy individual of same age to confirm their stature. Height is plotted on Center for Disease Control and Prevention (CDC) charts, used globally and a height that is two standard deviations below the mean height for age and sex (less than the 3rd percentile) is considered as short stature (Barstow and Rerucha, 2015). Complete skeletal survey through x-ray was performed which showed dysostosis multiplex, fish mouth vertebrae and bullet shaped metacarpals in Morquio syndrome patients.

### Sampling and DNA extraction

Peripheral blood samples of 3–5 mL were collected from 58 individuals from 14 families, covering 26 affected and 32 unaffected individuals. The blood samples were collected in 10 mL EDTA vacutainers (BD vacutainer K2 EDTA 18 mg). DNA extraction was performed using the Phenol-Chloroform method and DNA was quantified using a μDrop Plate reader (Multiskan™, Thermo Fisher Scientific, and Waltham, MA, United States).

### Whole genome sequencing and variant prioritization

Whole genome sequencing, reads mapping, genotype calling, pre-analysis quality assurance, and annotation were performed as describe previously by Gul et al. (2023).

Briefly, mapping and genotype calling was performed using a modified version of the PALEOMIX pipeline (https://pubmed.ncbi.nlm.nih.gov/24722405/), with initial quality assurance performed using FastQC (https://www.bioinformatics.babraham.ac.uk/projects/fastqc/) and MultiQC (https://pubmed.ncbi.nlm.nih.gov/27312411/). Reads were processed using fastp (https://pubmed.ncbi.nlm.nih.gov/30423086/) and mapped against the hg38 human reference using BWA (https://arxiv.org/abs/1303.3997). Alignments were post-processed using samtools (https://pubmed.ncbi.nlm.nih.gov/33590861/) and ‘bwa-postalt.js’ from the BWA-kit. BAMs were calibrated, and genotypes were called and calibrated using GATK (https://pubmed.ncbi.nlm.nih.gov/20644199/). The resulting VCF was annotated using VEP (https://pubmed.ncbi.nlm.nih.gov/27268795/).

Initially, analysis of variants identification was based on MPS-associated genes. Variants in the exomes were extracted from whole genome sequencing data of all individuals. The variants including non-synonymous and splice sites having minor allele frequency (MAF) greater than 0.01 in larger outbred populations were excluded by using gnomAD. In pedigrees with more than one affected individual, variants shared by multiple patients were prioritized. Based on consanguinity and inheritance pattern, homozygous variants were selected and prioritized. During re-analysis, heterozygous variants were also screened for compound heterozygosity. The prioritization was based on segregation, gene function, association with disease and pathogenicity prediction according to ACMG guidelines and *in silico* analysis (Richards et al., 2015). Genes were annotated according to the following transcripts: NM_000263.4 (*NAGLU*); NM_144992 (*VWA3B*); NM_000199.5 (*SGSH*); NM_000512.5 (*GALNS*); NM_001281723.2 (*BTD*); NM_032520.5 (*GNPTG*).

All variants were found in homozygous state in patients while in heterozygous state in parents. Phenotypically normal siblings in the present families were either homozygous wild type or heterozygous carriers for that particular variant. All identified variants are listed in Table 2 and Table 4. The variants were evaluated using ACMG guidelines and were predicted as pathogenic, likely pathogenic or uncertain significance. To exclude non-disease associated variants, the genomes of more than 100 ethnically matched *in-house* control samples were screened. The analysis plan for this study was same as mentioned in previously (Gul et al., 2023).

**TABLE 2:** *In-silico* analysis and ACMG classification of the identified variants in suspected Sanfilippo syndrome cases.

Pedigree/Patient ID	Variants	Patient genotype	dbSNP	In-silico tools with deleterious output	ACMG classification	VarSome	HGMD accession number	ClinVar accession numbers
A/VII-4	NM_000263.4 (NAGLU), c.2020C>T (p.Arg674Cys)	Homozygous	rs763299645	9/9	Pathogenic PP5, PP3, PM5, PM2	Pathogenic	CM981369	SCV004012870
B/VII-2, VII-3, VII-5	NM_000199.5 (SGSH), c.221C>T (p.Arg74His)	Homozygous	rs778336949	9/9	Pathogenic PP5, PS3, PM5, PP3, PM2	Pathogenic	CM971354	SCV004012871
C/V-1, V-4	NM_144992 (VWA3B), c.2951A>C (p.Glu984Ala)	Homozygous	N/A	8/9	Likely benign BP1, BP4, PM2	Uncertain significance	Current study	SCV004012872
D/IV-1, IV-2	NM_000199.5 (SGSH), c.364C>T (p.Gly122Arg)	Homozygous	rs761607612	9/9	Pathogenic PP5, PP3, PM2	Pathogenic	CM971358	SCV004012873
E/VI-1, VI-2	NM_000199.5 (SGSH), c.258A>C (p.Asn86Lys)	Homozygous	N/A	8/9	Uncertain significance PM2	Uncertain significance	Current study	SCV004012874
F/IV-1, IV-2	NM_000199.5 (SGSH), c.817G>A (p.Asp273Asn)	Homozygous	rs1046551417	7/9	Likely pathogenic PP3, PM2, PP5	Likely pathogenic	CM002397	SCV004012875

BP, possibly benign; N/A, not available; PP, possibly pathogenic; PM, moderate pathogenic; PS, strong pathogenic.

### Protein modeling and protein-protein interaction

To generate the complete three-dimensional (3D) structures of *BTD, SGSH, VWA3B, GNTGP*, *GALNS, and NAGLU* proteins I-TASSER (Iterative Threading Assembly Refinement) (https://zhanggroup.org/I-TASSER/) was utilized. This is a bioinformatics tool that generates 3D protein structures using hierarchical techniques, first searching the PDB library for templates and then building the models using iterative template fragment assembly simulation. The degree of appropriateness of all structures were verified through VERIFY3D and ERRAT. UCSF Chimera software 1.14 was used to inspect all the structures graphically (Supplementary Figure S1).

### Evolutionary conservation analysis

The degree of evolutionary conservation of the amino acids was calculated using the ConSurf (https://consurf.tau.ac.il/consurf_index.php) server to maintain the structural integrity and function of protein, yielding a conservation rating from 1-9, classified as variable, intermediate, or highly conserved protein site. Furthermore, properties of residues were categorized as either exposed (e), buried (b), highly conserved and exposed (functional, f), or highly conserved and buried (s) (Supplementary Figure S2). As a result, any change in the highly conserved area has a significant impact on the protein’s integral structure and function.

## Results

### Clinical characterization

Based on clinical and phenotypic features, 11 patients (6 males and 5 females) with average age of ±7.2 years from six families were diagnosed with Sanfilippo syndrome (MPS III). The other 15 patients (9 males and 6 females) from 8 families were labeled as suspected cases of MPS IV with average age of ±7.3 years. MPS III patients showed a wide range of overlapping features like delay in achieving milestones, aggressive behavior, abdominal distention, increased body hair growth and intellectual disability (Table 1). MPS IV patients showing overlapping features like short stature, coarse facial features, scoliosis/kyphosis, gibbus formation and many others (Table 3). All families of MPS III show parental consanguinity except family D having intra-cast marriage without any direct blood relation among parents (Figure 1D). For MPS IV all families show consanguinity except family C (Figure 2C).

**TABLE 3:** Clinical details of the patients suspected as Morquio syndrome affected.

Pedigree no.	Individual no.	Affected individuals	Age/Sex	Cast/region/Consanguinity	Phenotypes
**A**	IV-1	1	3 y/M	Gujjar/Punjab/Yes	Delay in achieving milestones, abdominal distention, gibbus formation, short stature, height 95 cm, 12 kg weight, head circumference 48 cm, coarse facial features, broad based gait, joint contractures, kyphosis/scoliosis, bullet shaped metacarpals, beaking of vertebrae, oar shaped ribs, widening of medullary canal, coarse trabecular pattern Metaphyseal dysplasia, Blood sugar Random = 70 mg/dL (80–160), Hb = 11.7 g/dL (14–18), HCT = 34.3% (35–55), Neutrophils = 39.8% (60–70), Lymphocytes = 42.1% (30–40), Vitamin D3 = 18 ng/mL (Deficient <20)
**B**	IV-4	5	5 y/M	Awan/Punjab/Yes	Abdominal distention, aggressive behavior, short stature, constipation, (89 cm height, 14 kg weight, head circumference 51 cm)
	IV-5		7.5 y/F		Speech delay, gibbus formation, short stature (F = 92 cm height, weight 15 kg, head circumference 49 cm, coarse facial features, broad based gait, bone pain, joint contractures, short trunk, scoliosis/kyphosis, pectus carinatum
**C**	III-1	2	12 y/M	Pakhtoon/KPK/No	Asthma (uses inhaler), sleep apnea (wakes up suddenly), no hearing, very loose joints, thin bones, underweight, deep voice, macrocephaly, carpel tunnel syndrome, short stature, spinal cord is bent, all body muscles and bones deform, weak muscles, aggressive, coarse facial features, jerky movement, widely spaced teeth, large tongue, mild ID, flat nasal bridge, skin problem
	III-2		9 y/F		Sleep apnea (wakes up suddenly), very loose joints, thin bones, underweight, deep voice, macrocephaly, short stature, spinal cord is bent, all body muscles and bones deform, weak muscles, aggressive, coarse facial features, jerky movement, widely spaced teeth, large tongue, mild ID, flat nasal bridge
**D**	IV-1	3	10.5 y/M	Mohmand/KPK/Yes	Delay in achieving milestones, abdominal distention, aggressive behavior, gibbus, short stature, respiratory/ear infection, coarse facial features, joint contractures, short trunk, scoliosis/kyphosis, hernia, broad based gait
	IV-2		9 y/F		Delay in achieving milestones, abdominal distention, aggressive behavior, gibbus, short stature, respiratory/ear infection, coarse facial features, joint contractures, short trunk, scoliosis/kyphosis, hernia, broad based gait
	IV-3		8 y/F		Delay in achieving milestones, abdominal distention, aggressive behavior, gibbus, short stature, respiratory/ear infection, coarse facial features, joint contractures, short trunk, scoliosis/kyphosis, hernia, broad based gait
**E**	VI-1	2	10 y/M	Qureshi/Punjab/Yes	Aggressive behavior, short stature, gibbus formation, waddling gait, joint contractures, short trunk, scoliosis/kyphosis, liver problem, anemia, sleep apnea
	VI-2		8 y/F		Aggressive behavior, short stature, gibbus formation, waddling gait, joint contractures, short trunk, scoliosis/kyphosis, liver problem, anemia, sleep apnea
**F**	IV-1	1	13 y/F	*Rana*/AJK/Yes	Short stature, gibbus formation, joint contractures, short trunk, scoliosis/kyphosis, coarse facial features, spondyloepiphyseal dysplasia, height = 80cm, weight = 10 kg
**G**	IV-3	3	4 y/M	Awan/KPK/Yes	Speech delay, abdominal distention, short stature, gibbus formation, respiratory/ear infection, coarse facial features, short trunk, joint contractures, scoliosis/kyphosis, hernia, hepatosplenomegaly
	IV-5 IV-6		3 y/M 3 y/M		Speech delay, abdominal distention, short stature, gibbus formation, respiratory/ear infection, coarse facial features, short trunk, joint contractures, scoliosis/kyphosis, hernia, hepatosplenomegaly (twins)
**H**	V-3	1	5 y/M	Pakhtun/KPK/Yes	Delay in achieving milestones, short stature, coarse facial features, scoliosis/kyphosis, abdominal distention, hepatosplenomegaly, joint contractures, beaking of chest Hb = 11.8 g/dL (12–14), Bilirubin = 16.4 mg/dL (0.1–1.0), Serum growth hormone = 1.62 ng/mL (2–5)

AJK, azad jammu and kashmir; cm, Centimeter; F, female; Hb, Hemoglobin; HCT, hematocrit; kg, Kilogram; KPK, Khyber Pakhtunkhwa; M, male; y, years.

### Genetic characterization

Among 14 families diagnosed with MPS, 6 novel and 6 reported variants were identified, where 11 families carried variants in MPS-associated genes (*NAGLU, SGSH, GALNS*), two families carried variants in two genes (*VWA3B, GNPTG*) that were not previously associated with MPS phenotypes, while one family remained genetically undiagnosed. In family H we found dual molecular diagnosis (*GALNS* and *BTD* genes involvement). In family C (MPS IV), initial analysis of coding regions in patients did not reveal any homozygous or compound heterozygous pathogenic variant(s).

## *In silico* analysis

### Pathogenicity prediction

The identified variants were predicted to be deleterious/pathogenic by various mutation prediction tools like MutationTaster, I-TASSER, SIFT, Polyphen-2, CADD, VarSome, VARSEAK and PROVEAN (Table 2; Table 4). Generated structures were selected based on their high C-score and Tm-score. Wild-type structure of proteins (*BTD*, *SGSH*, *VWA3B*, *GNPTG*, *NAGLU*, and *GALNS*) were retrieved from Alpha-fold. Comparison between the wild-type and mutant type was performed and the mutated structures showed significant structural change. The altered structure may result in a loss of function, altered interactions with ligands or neighboring proteins, or altered stability of the proteins. None of the identified variants were found in homozygous state in any human genome variation databases including ExAC, gnomAD, 1000 Genome Project, dbSNP and healthy control samples of same ethnicity.

**TABLE 4:** In-silico analysis and ACMG classification of the identified variants in suspected Morquio syndrome cases.

Pedigree/Patient ID	Variants	Patient genotype	dbSNP	In-silico tools with deleterious output	ACMG classification	VarSome	HGMD accession number	ClinVar accession numbers
A/IV-1	NM_000512.5 (*GALNS*), c. 1365-2A>G	Homozygous	rs1281515662	N/A	Likely pathogenic PVS1, PM2	Likely pathogenic	Current study	SCV004012876
B/IV-4,IV-5	NM_000512.5 (*GALNS*), c.107A>C (p.Leu36Arg)	Homozygous	rs755832705	8/9	Pathogenic PP5, PM1, PM5, PP3, PM2	Pathogenic	CM145571	SCV004012877
C/III-1,III-2	Unsolved	Homozygous	N/A	N/A	N/A	N/A	N/A	N/A
D/IV-1,IV-2,IV-3	NM_000512.5 (*GALNS*), c.1259G>C (p.Pro420Arg)	Homozygous	N/A	9/9	Uncertain significance PP3, PM2, PP5	Uncertain significance	CM145597	SCV004012878
E/VI-1,VI-2	NM_032520.5 (*GNPTG*), c.179G>A (p.Gly60Glu)	Homozygous	N/A	8/9	Uncertain significance PP3, PM2	Uncertain significance	Current study	SCV004012879
F/IV-1	NM_000512.5 (*GALNS*), c.423-1G>A	Homozygous	N/A	N/A	Pathogenic PVS1, PP5, PM2	Pathogenic	Current study	SCV004012880
G/IV-3/IV-5,IV-6	NM_000512.5 (*GALNS*), c.107A>C (p.Leu36Arg)	Homozygous	N/A	8/9	Pathogenic PP5, PM1, PM5, PP3, PM2	Pathogenic	CM145571	SCV004012877
H/V-3	NM_000512.5 (*GALNS*), c.107A>C (p.Leu36Arg)	Homozygous	N/A	8/9	Pathogenic PP5, PM1, PM5, PP3, PM2	Pathogenic	CM145571	SCV004012877
	NM_001281723.2 (*BTD*), c.1336G>C (p.Asp446His)	Homozygous	N/A	8/9	Pathogenic PS3, PS1, PP5, PM1, PM5	Pathogenic	Current study	SCV004012883

N/A, not available; PP, possibly pathogenic; PM, moderate pathogenic; PS, strong pathogenic, PVS, very strong pathogenic.

### Evolutionary conservation analysis

The evolutionary conservation analysis of candidate genes showed that, in BTD protein, p.Asp446His is an average (partially conserved) and exposed residue according to the NACSES. In the protein SGSH, the Asn86Lys is highly conserved and buried residue. In protein VWA3B*,* the Glu984Ala is highly conserved, exposed and functional residue. In protein GNPTG, the Gly60Glu is highly conserved, buried and structural residue according to NACSES. In the protein *NAGLU,* Arg674Cys is highly conserved, exposed and functional residue according to NACSES.

## Discussion

In 14 families with phenotypically diagnosed MPS III (A-F) or MPS IV (A-H) we report that 11 families carried pathogenic variants in known MPS-associated genes. Two families had novel mutations in genes previously not associated with MPS and one family remains genetically undiagnosed. Additionally, one family (family H) had pathogenic variants in both *GALNS* and *BTD* segregating with the phenotype.

### Mucopolysaccharidoses III

The initial recruitment and diagnosis of six families with Sanfilippo syndrome (MPS III) were based on their clinical profiles and phenotypes (Table 1).

We report two likely causal variations: One identified in *NAGLU* (family A) co-segregating with MPS III type B and the other in *SGSH* (families B, D, E and F) co-segregating with MPS III type A and *VWA3B* (Family C).

In family A, a homozygous missense variant c. 2020C>T (p. Arg674Cys) was found in exon 6 of *NAGLU* gene. This variant was first reported in compound heterozygous state by Zhao et al. (1998) in two different families affected with end-stage neurological debilitation and moderate mental retardation. Another missense variant at the same codon (c. 2021G>A (p. Arg674His)) in compound heterozygous state was reported by Zhao et al. (1996) in an Arab family. This locus is the mutational hot spot for substitution of Arginine. The locus has CpG dinucleotide involvement (Zhao et al., 1996). Currently studied patient showed more severe phenotypes including delay in achieving milestone, abdominal distention, aggressive behavior, speech delay, short stature, increased body hair, coarse facial features, broad-based gait, joint contracture, short trunk, hepatosplenomegaly, self-biting and intellectual disability. The interfamilial variability in the severity of phenotypes might be due to homozygous substitution of Arginine at amino acid position 674 in the present patient and/or different ethnicities of the families reported by Zhao et al. (1996), Zhao et al. (1998). Furthermore, such variability in clinical presentations amongst patients harboring the same genetic variant have also been reported for other LSD subtypes, indicating the contribution of epigenetic modifications, environmental factors and/or genetic modifiers that is yet to be elucidated (Davidson et al., 2018; Sheth et al., 2022).

In family B, five individuals were affected including three males and two females. One female (VI-1) and one male (VII-1) patient had died at the age of 12 and 14 years respectively. All available patients were homozygous for the *SGSH* c. 221C>T (p. Arg74His) variant. The parents were heterozygous, while two healthy live siblings including a female (VII-5; 21 years) and a male (VII-4; 15 years) were wild type carriers. This pathogenic variant was first identified in compound heterozygous form in the Polish population (Bunge et al., 1997). The same codon is altered for another substitution where Arginine is replaced by Cysteine (c. 222G>A, p.Arg74Cys) (Bunge et al., 1997) resulting in a similar phenotype. This locus is likely to be the mutational hot spot for the disease. This residue is conserved among all mammalian sulfatases and stabilizes the active site of the enzyme (Bunge et al., 1997). The substitution might cause loss of ionic interactions formed by positively charged arginine; also, difference in sizes causes loss of interactions with other residues and domains.

In family C, a novel *VWA3B* variant (c.2951A>C; p.Glu984Ala, exon 22) was identified. The variant causes loss of interactions and loss of hydrogen bonds thus affecting the correct folding of protein (HOPE analysis https://www3.cmbi.umcn.nl/hope/).

Previously, four variants including three nonsense and one missense have been reported in *VWA3B* underlying neurological phenotypes including intellectual disability, cerebellar ataxia and autism spectrum disorder as per HGMD (Accessed on 20-May-2022). Affected individuals in the present study showed MPS III overlapping phenotypes including delay in achieving milestones, chronic diarrhea, aggressive behavior, speech delay, short stature, coarse facial features, joint contractures, hepatosplenomegaly, and intellectual disability. Variability in the phenotypes of previous reported cases and the patients in the present study might be due to different position of the variant, familial background, and/or the impact of SNPs in modifier genes.

In family D, the homozygous missense variant *SGSH* c. 364C>T (p. Gly122Arg) in exon 4 was associated with MPS III phenotype in both patients. This variant was first reported in 1997 in Dutch and Arab patients (Bunge et al., 1997). Insertion of a charged residue Arginine at amino acid position 122 of SGSH will cause distortion in the structure and might cause disruption of correct folding of protein. Clinical details and pathogenicity of the variant is described in Table 1 and Table 2, respectively. There were two affected individuals in the family, one sister and one brother born to a non-consanguineous couple, without any previous family history for the disease but all the successive marriages in previous generations are intra-cast marriages (Figure 1D).

In family E, a homozygous missense *SGSH* c. 258A>C (p. Asn86Lys) variant was identified. This variant is novel and has not previously been reported to cause Sanfilippo syndrome. The family had two affected males, born to a first-degree cousin couple (Figure 1E). One patient VI-1 died a month after sample collection at the age of 15 years. Asparagine at amino acid 86 is highly conserved in SGSH in different orthologues. The wild-type residue forms a hydrogen bond with methionine at position 88, the difference in size of wild type and mutated residues causes loss of interaction. The variation is in the catalytic domain that is important for the activity of the enzyme and interact with another domain which might affect the function of protein. As per ACMG guidelines, the variant was classified as a variant of uncertain significance (Table 2).

In family F, the identified pathogenic variant was *SGSH* c. 817G>A (p. Asp273Asn) in exon 7. This variant was reported in UK in 2000 (Beesley et al., 2000). The variant was found in homozygous state in both patients (IV-1, IV-2) and heterozygous in their parents. Both affected individuals were females and born to a first-degree cousin couple (Figure 1F). This variant is located in a CpG dinucleotide site which is the mutational hot spot of the gene (Beesley et al., 2000). As per HOPE analysis, the wild-type residue is negatively charged, and therefore, interaction with calcium ions is lost with this substitution, reducing the stability of the enzyme. The wild type residue Asp273 also forms hydrogen bonds with Asp31 and Asp32 and salt bridges with Arg74, Leu123, and Arg282, which might be lost due to substitution of Asp273 with a neutral Asparagine residue, disturbing the ionic interactions. As per ACMG guidelines the variant is classified as likely pathogenic (Table 2).

Functional lysosomes are important for autophagy to regulate the quality of cytoplasm by eliminating cellular macromolecular aggregates. If autophagy is disturbed due to compromised or absent lysosomal enzyme it may lead to inappropriate *storage* of material in different cells. This storage interferes with normal cell function and affects many organ systems including brain, viscera, bone and cartilage causing various diseases including lysosomal storage disorders (LSDs), neurodegenerative diseases and cancers (Saha et al., 2018; Scerra et al., 2022). Interestingly, in present study we identified missense disease-causing variants in all MPS-III diagnosed families, such variants could have little or no impact on the enzymatic activity of the mutant protein but may cause folding or tertiary structure alterations. Misfolded protein/s are retained in the endoplasmic reticulum for subsequent degradation thus causing enzyme deficiency and compromise in autophagy (Mohamed et al., 2017; Valencia et al., 2021). Therefore, neurological symptoms are one of the most common phenotypes observed in MPS III because neurons being post-mitotic cells could not dilute damaged cell organelles and protein aggregates (Valencia et al., 2021; Scerra et al., 2022). No therapeutic approach has been successful in reverting the symptoms of MPS III and neurological manifestations caused by it. Some of the common therapies tested include enzyme replacement therapy (ERT), substrate reduction therapy (SRT), pharmacological chaperon therapy (PCT) and hematopoietic stem cell transplant (HSCT) (Valstar et al., 2010; Beneto et al., 2020; Penon-Portmann et al., 2023). Some of the therapies applying on cell lines and animal models for treating MPS III include over expression of TFEB (Lotfi et al., 2018), master regulator of lysosomal biogenesis (Bajaj et al., 2019) and coenzyme Q_10_ (Matalonga et al., 2014).

### Mucopolysaccharidoses IV

Eight families (A-H) were recruited on the basis of their MPS IV (Morquio syndrome type A) phenotypes. In six families (A, B, D, F, G, H) identification of *GALNS* mutations confirmed the diagnosis. In family E, a variant in the mucolipidosis-associated gene *GNPTG* segregated with the phenotype. No disease-causing variant could be identified in family C.

In family A, the patient had a novel homozygous *GALNS* splice site variant g.38845A>G (chr16:88884534T>C); c.1365–2A>G (intron 12). A splice site prediction tool varSEAK (https://varseak.bio/) predicted the identified splice site variant with class 5 (splicing effect). The identified variant is predicted to activate a cryptic site 34 nucleotide upstream of 3′splice site leading to the production of an abnormal protein.

Three families B, G and H shared a highly prevalent *GALNS* c.107T>G; p.Leu36Arg missense variant. Previously, two different variations (c. 107T>G; p. Leu36Arg; c. 107T>C, p.Leu36Pro) were identified on the same codon to cause Morquio syndrome type A in Asian-multiethnic and Mexican populations respectively (Tomatsu et al., 2005; Morrone et al., 2014). The variation (p.Leu36Arg) is in the catalytic domain, which can disturb the function of the enzyme. It may also hinder the correct protein folding due to the charged arginine residue leading to the loss of hydrophobic interactions. All the three families in the present study showed consanguinity and homozygosity of the variant in patients, while parents were heterozygous carriers (Figures 2B,G,H). Clinical examination demonstrated intra and interfamilial variability in the phenotypes of patients carrying the same variant (Table 3). The variability of phenotypes in this study might be due to different ethnicities of affected families. Additionally, family H showed dual molecular diagnosis as another homozygous missense novel variant in *BTD* gene, c. 1336G>C; p. Asp446His causing biotinidase deficiency (EC 3.5.1.12) segregated with the phenotype. This enzyme is involved in the recycling of biotin bound to protein by releasing biotin and lysine (Pispa, 1965; Wolf et al., 1985). The wild-type residue Asp at 446 forms hydrogen bonds with Gly423, Tyr456 and Gln458, and a salt bridge with Arg544, but because of the substitution, hydrogen bonding and ionic interaction will be lost.

In family D, there were three affected individuals, two females and a male born to first-degree cousin parents (Figure 2D). A homozygous *GALNS* c. 1259G>C; p. Pro420Arg missense variant segregated with disease. Previously, Morrone et al. (2014) identified the same variant to cause MPS IV in Asian-multiethnic population. Ullah et al. (2017) reported a consanguineous Pakistani family affected with MPS IV segregating same disease-causing variant, i.e., p. Pro420Arg where substitution of proline residue with arginine distorts the protein conformation (Ullah et al., 2017).

In family E, a novel homozygous *GNPTG* c.179G>A; p.Gly60Glu missense variant was identified. Variants in *GNPTG* cause mucolipidoses type III (ML III). This gene encodes the gamma subunit of enzyme N-acetylglucosamine-1-phosphotransferase (EC 2.7.8.17) responsible for catalyzing the first step in synthesis of a mannose 6-phosphate lysosomal recognition marker (Braulke et al., 2008; Qian et al., 2010). The clinical profile for ML III includes phenotypes like short stature, scoliosis and joint contractures (Tiede et al., 2005; Bargal et al., 2006), which were also present in the patients in the present study (Table 3). As the initial diagnosis in the present study was based on clinical phenotypes, and the two disorders (MPS and ML) have overlapping phenotypes, we could not rule out the possibility of dual molecular diagnosis. Next-generation sequencing techniques should be used for the correct diagnosis of these complex overlapping phenotypes. Glycine at amino acid 60 in the GNPTG protein is highly conserved, so its substitution is probably damaging to the protein. The mutant residue is negatively charged so its incorporation will lead ligands and other residues of same charge to be repelled.

In family F, there were two affected individuals, one living while the other died at the age 14. A novel homozygous splice acceptor site *GALNS* c.423-1G>A variant was found in the patient. The parents were first degree cousins (Figure 2F). The identified variant is predicted to activate a cryptic site 1 nucleotide downstream of 3′splice site leading to a frameshift mutation (varSEAK Online - Splice Site Prediction Version 2.1).

Therapies for MPS IV include enzyme replacement therapy which is currently working only for type A not for type B. Some therapies in experimental phases include substrate reduction therapy, hematopoietic stem cell transplant, gene therapy, anti-oxidant therapy, inhibiting protein aggregation, stope codon read through therapy and anti-inflammatory therapy (Akyol et al., 2019; Seker Yilmaz et al., 2021; Penon-Portmann et al., 2023). In a recent therapy, the delivery of recombinant human GALNS enzyme (rhGALNS) into fibroblasts was achieved by infusing it with a hydrogel, such as polyethylene glycol. This innovative approach ensures sustainable and prolonged release of the exogenous enzyme. However, it is important to consider that this treatment might be costly and require frequent administration (Jain et al., 2020).

From Pakistan, very little knowledge about the genetic basis of MPS IV has revealed a common variant p.Leu36Arg in 3/8 families sequenced during current study, and another variant p.Pro420Arg reported formerly by Ullah et al., 2017, also found in family D with three affected siblings.

In Pakistan, the burden of recessively inherited lethal genetic disorders like Sanfilippo and Morquio syndromes is higher due to custom of intra-familial marriages, but the precise diagnosis is lacking due to unavailability of state-of-the-art diagnosis facilities (Hussain and Bittles, 1998; Shahid et al., 2020; Shahzadi et al., 2020; Gul et al., 2023). However, to ensure the health of children suffering from such life-threatening disorders, timely diagnosis and treatment is essential. Patients without proper medical care are affected by multiple medical conditions and progressive tissue degeneration. Therefore, early, and correct diagnosis of such disorders should be performed using targeted gene sequencing and/or WGS. Furthermore, molecular genetic diagnosis will help to provide premarital carrier diagnosis, genetic counselling and to devise prenatal screening tests to address this deficit in the foreseeable future.

**Strength of Study:** This study details extensive work from a highly consanguineous population of Pakistan, showing the need of genetic exploration for more detailed analysis related to lethal genetic disorders like Sanfilippo and Morquio syndromes.

**Limitation of Study:** The initial diagnosis of the patients included in this study, was performed based on clinical and radiographic examinations; thus, misdiagnosis of the patients could not be ruled out due to overlapping phenotypes of MPS, *VWA3B*-related phenotypes and ML. Furthermore, the effect of the identified variants on protein structure and function is based on *in silico* tools, and future functional studies are needed to validate the prediction/s.

## Data Availability

The datasets presented in this study can be found in online repositories. The names of the repository/repositories and accession number(s) can be found in the article/Supplementary material.

## Supplementary material

SUPPLEMENTARY FIGURE S1 Three-dimensional (3D) of wild-type and mutants of *BTD, SGSH, VWA3B, GNTGP*. The zoom-in part represents the mutated residue of each protein. Mutated proteins show a significant change in their structure.

SUPPLEMENTARY FIGURE S2 Evolutionary conservation of mutated residue of *BTD, SGSH, VWA3B,* and *GNTGP*. The red boxes represent wild-type amino acids that might be altered by pathogenic mutations of high risk. The conservation scale has a score range of 1–9, with conserved sites receiving ratings in the range of 7–9. The tool illustrated the properties of residue that is either exposed (e), buried (b), highly conserved and exposed (functional, ,**(F)** or highly conserved and buried (b).
