# Supplementary figures and images for: Identification of genetic variants associated with a wide spectrum of phenotypes clinically diagnosed as Sanfilippo and Morquio syndromes using whole genome sequencing
Source: Front Genet. 2023 Sep 11;14:1254909. doi: 10.3389/fgene.2023.1254909 (PMC10524275; doi:10.3389/fgene.2023.1254909)

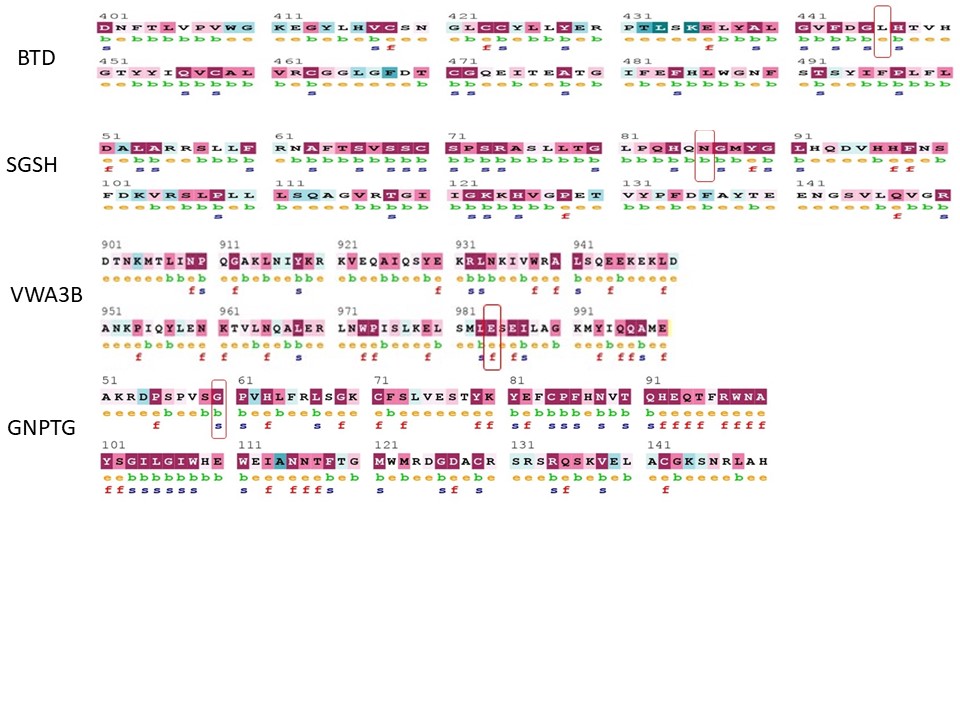

Supplement: Supplementary file 1 [file Image1.jpeg]

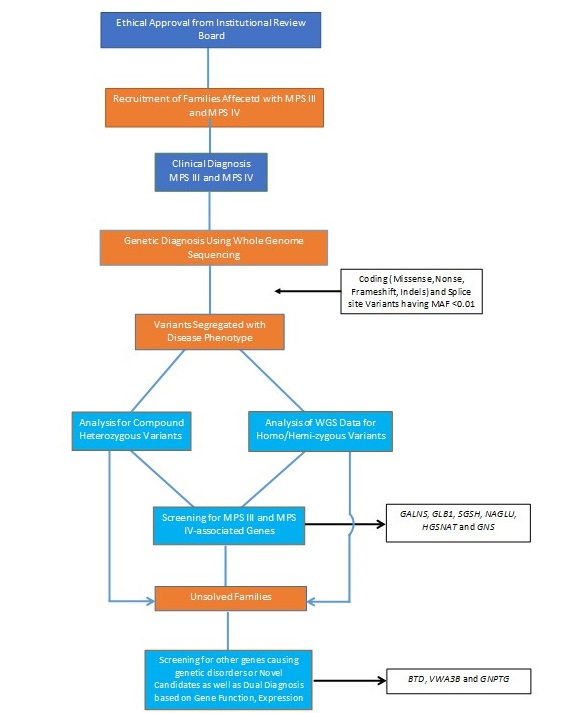

Supplement: Supplementary file 2 [file Image2.jpeg]
